# Supplementary material for: Functional recruitment and connectivity of the cerebellum is associated with the emergence of Theory of Mind in early childhood
Source: Nat Commun. 2025 Jun 6;16:5273. doi: 10.1038/s41467-025-60523-9 (PMC12144182; doi:10.1038/s41467-025-60523-9)
Supplement: Supplementary file 1 — Supplementary Information [file 41467_2025_60523_MOESM1_ESM.pdf]

Supplementary Information

Functional recruitment and connectivity of the cerebellum is associated with the emergence of Theory of Mind in early childhood

Aikaterina Manoli[1,2,3,4,5], Frank Van Overwalle[6,7], Charlotte Grosse Wiesmann[3,8]*, Sofie L. Valk[2,4,9]*

[1] International Max Planck Research School on Cognitive Neuroimaging (IMPRS CoNI), Max Planck Institute for Human Cognitive and Brain Sciences, Leipzig, Germany

[2] Lise Meitner Group Neurobiosocial, Max Planck Institute for Human Cognitive and Brain Sciences, Leipzig, Germany

[3] Minerva Fast Track Group Milestones of Early Cognitive Development, Max Planck Institute for Human Cognitive and Brain Sciences, Leipzig, Germany

[4] Institute of Neuroscience and Medicine, Brain & Behavior (INM-7), Research Centre Jülich, Jülich, Germany

[5] Faculty of Medicine, Leipzig University, Leipzig, Germany

[6] Brain, Body and Cognition Research Group, Faculty of Psychology and Educational Sciences, Vrije Universiteit Brussel, Elsene, Belgium

[7] Center for Neurosciences (C4N), Vrije Universiteit Brussel, Elsene, Belgium

[8] Cognitive Neuroscience Lab, Department of Liberal Arts and Sciences, University of Technology Nuremberg, Germany

[9] Institute of Systems Neuroscience, Medical Faculty, Heinrich Heine University Düsseldorf, Düsseldorf, Germany

* = Shared last authorship

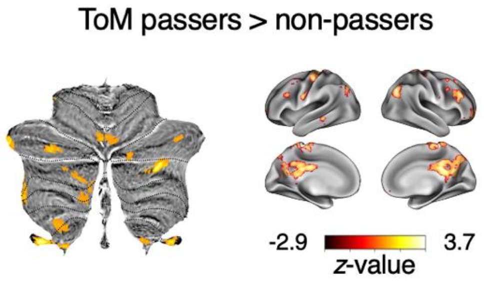

Supplementary figure 1. Functional activation controlling for IQ. General linear model (GLM) of activation differences as a function of children's ToM task performance (0-6), accounting for IQ score (WPPSI or KBIT-II) (two-sided; *z*-scored and FDR-corrected at *q* = .05; *N* = 41).

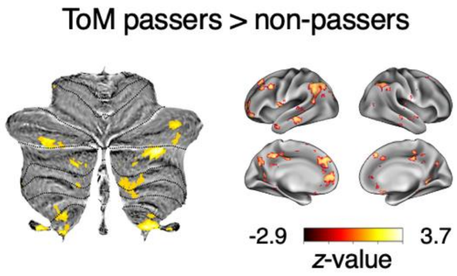

Supplementary figure 2. Functional activation controlling for age and sex. GLM of activation differences as a function of children's ToM task performance (0-6), accounting for age and sex (two-sided; *z*-scored and FDR-corrected at *q* = .05; *N* = 41).

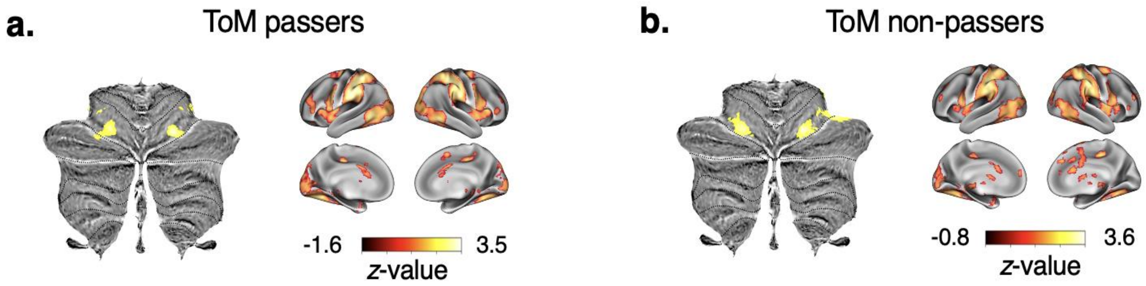

Supplementary figure 3. Functional activation during movie scenes depicting bodily pain as a function of children's ToM abilities (*z*-scored and FDR-corrected at q = .05). **a.** One-sample *t*-test showing activations for bodily pain vs. ToM movie scenes in ToM passers (N = 22). **b.** One-sample *t*-test showing activations for bodily pain vs. ToM movie scenes in ToM non-passers (N = 19). All analyses are two-sided.

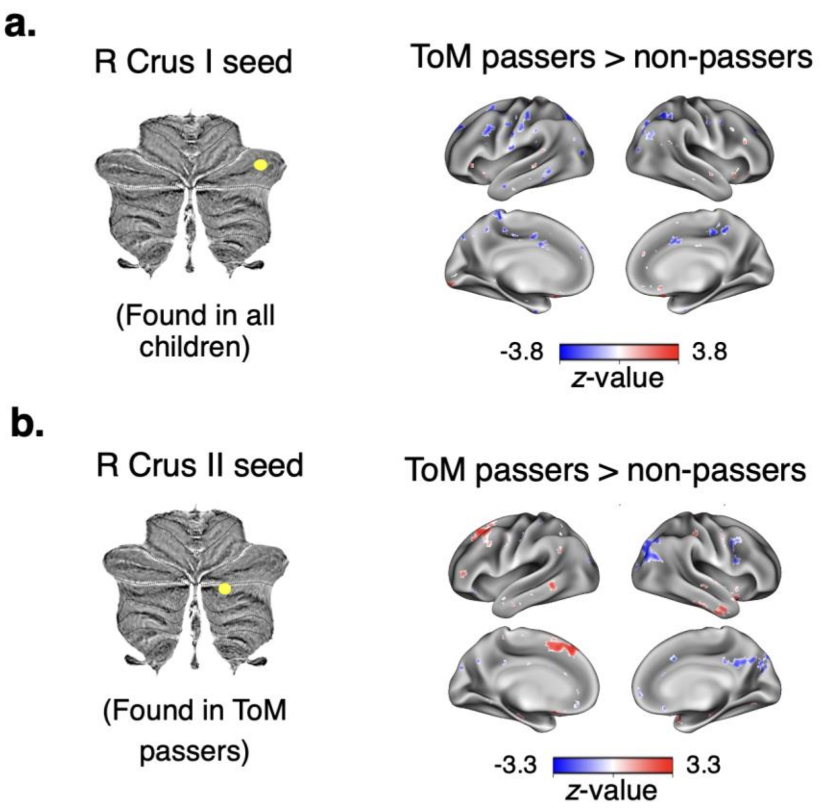

Supplementary figure 4. Seed-to-voxel functional connectivity between cerebellar ToM clusters and the cerebral cortex controlling for age and sex (*z*-scored and FDR-corrected at $q = .05$; $N = 41$). **a.** GLM of cerebro-cerebellar connectivity as a function of children's ToM abilities for rCrus I (cluster found in all children), accounting for age and sex. **b.** GLM of cerebro-cerebellar connectivity as a function of children's ToM abilities for rCrus II (cluster found in ToM passers), accounting for age and sex. All analyses are two-sided.

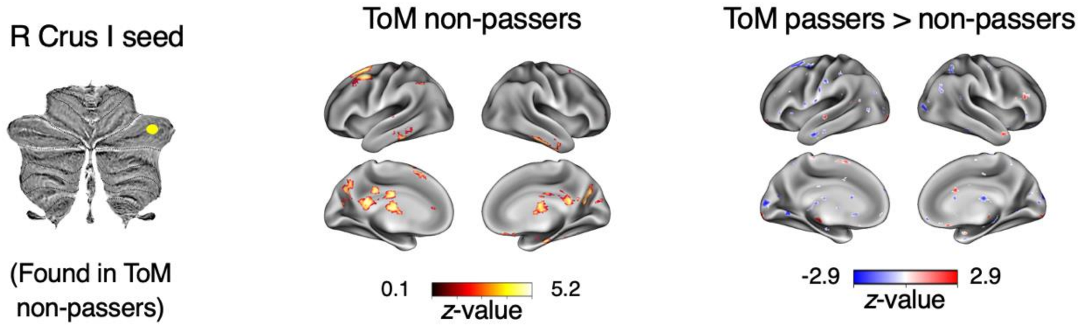

Supplementary figure 5. Seed-to-voxel functional connectivity between cerebellar ToM clusters and the cerebral cortex in ToM non-passers (*z*-scored and FDR-corrected at $q = .05$). Left: One-sample *t*-test of connectivity of rCrus I (cluster found in ToM non-passers) with the cerebral cortex in ToM non-passers ($N = 19$). Right: Two-sample *t*-test of connectivity differences of rCrus I between ToM passers and non-passers ($N = 41$). All analyses are two-sided.

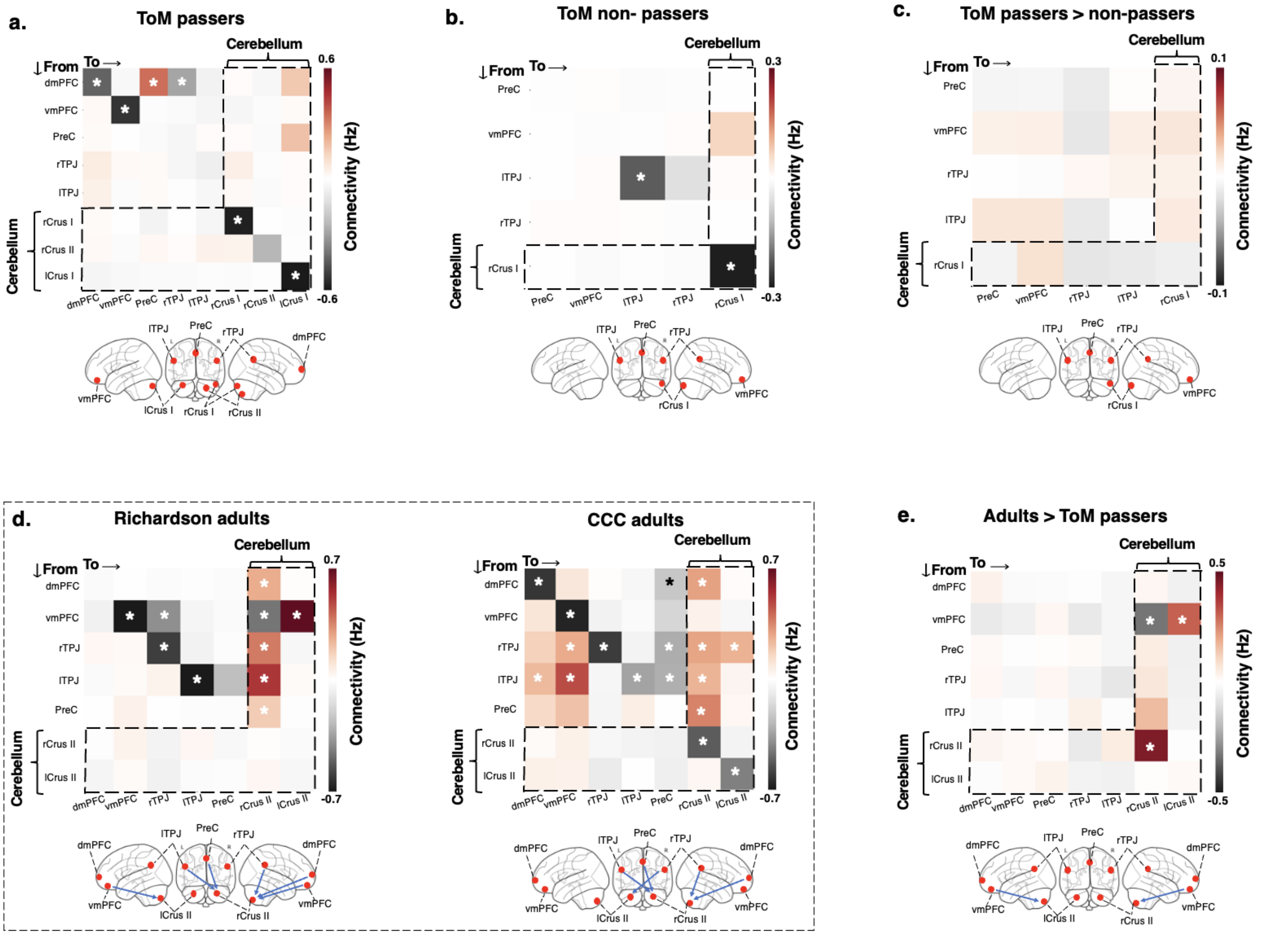

Supplementary figure 6. Dynamic causal modeling (DCM) of the cerebellum and the cerebral ToM network in children and adults. Averaged modulatory (task-dependent) connections in children and adults (in units of Hz). The vertical axis represents connections that originate from a seed region and terminate to a target region (represented in the horizontal axis). **a, b.** Modulatory connections in (a) ToM passers (*N* = 22) and (b) ToM non-passers (*N* = 19), using ToM activation clusters (identified via the in-scanner ToM task) as ROIs. **c.** Connectivity differences between ToM passers and non-passers (*N* = 41), identified by adding group type (ToM pass, ToM fail) as a covariate in the model. **d.** Modulatory connections in adults in the Richardson et al. (2018) (*N* = 22) and Caltech Conte Center samples (*N* = 56) using ToM ROIs identified in a functional atlas of ToM activations in adults (King et al., 2019). **e.** Connectivity differences between ToM passers and adults (in the Richardson et al., 2018 sample), using group type (adult, child) as a covariate (*N* = 44). Green arrows in the glass brains represent connections from the cerebellum to the cerebral cortex. Blue arrows represent connections from the cerebral cortex to the cerebellum. Glass brains were plotted with the Nilearn Python library. * = Bayesian posterior probability > .95. Abbreviations: CCC = Caltech Conte Center; dmPFC = dorsomedial prefrontal cortex; vmPFC = ventromedial prefrontal cortex; PreC = precuneus; r/lTPJ = right/left temporoparietal junction.

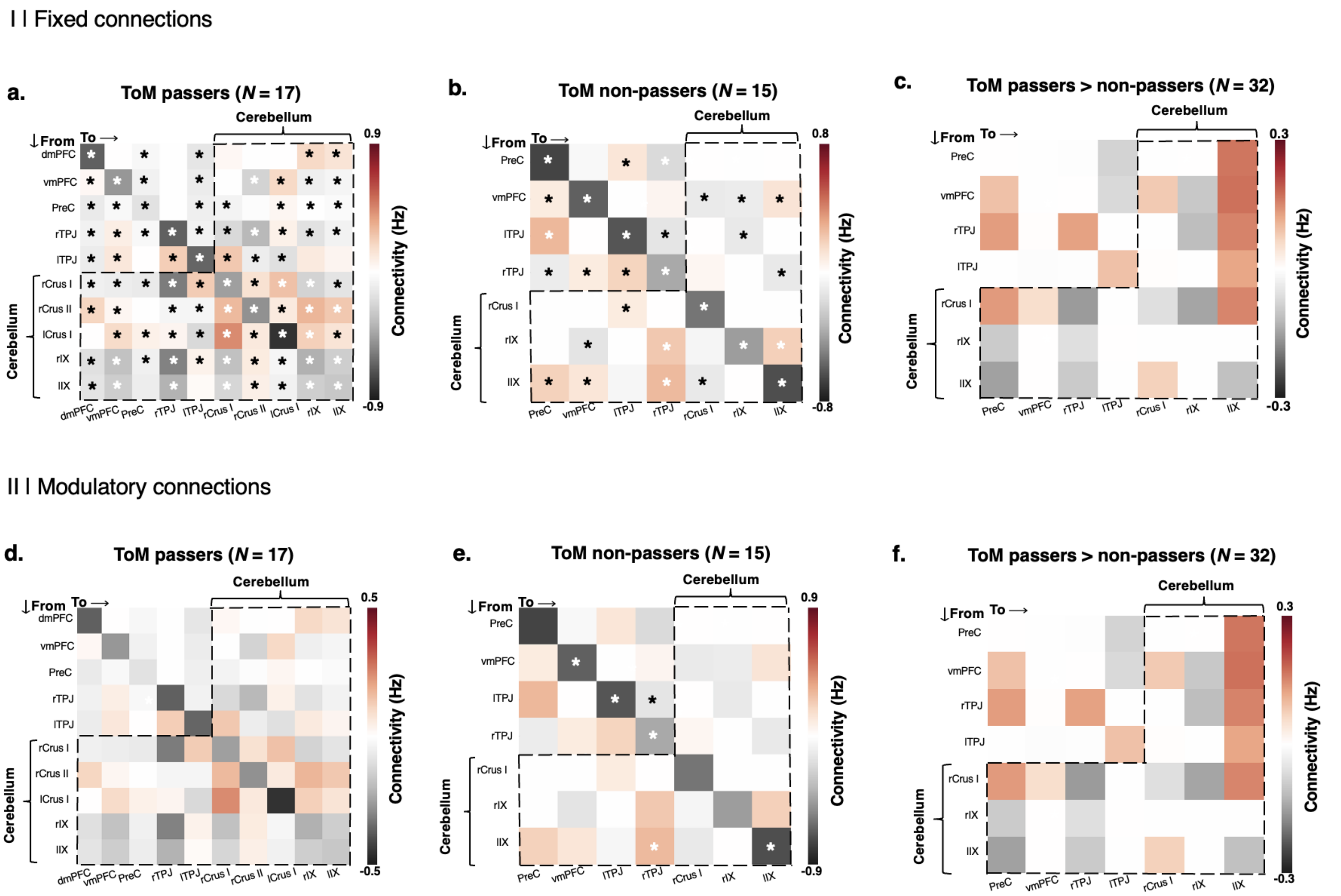

Supplementary figure 7. Dynamic causal modeling (DCM) of the cerebellum and the cerebral ToM network in a subset of children with activations in the bilateral IX. Averaged fixed (task-independent; I) and modulatory (task-dependent; II) connections in ToM passers and non-passers (in units of Hz). The vertical axis represents connections that originate from a seed region and terminate to a target region (represented in the horizontal axis). **a, b, d, e.** Modulatory connections in (a, d) ToM passers and (b, e) ToM non-passers, using ToM activation clusters (identified via the in-scanner ToM task) as ROIs. **c, f.** Connectivity differences between ToM passers and non-passers, identified by adding group type (ToM pass, ToM fail) as a covariate in the model. * = Bayesian posterior probability > .95. Abbreviations: dmPFC = dorsomedial prefrontal cortex; vmPFC = ventromedial prefrontal cortex; PreC = precuneus; r/lTPJ = right/left temporoparietal junction.

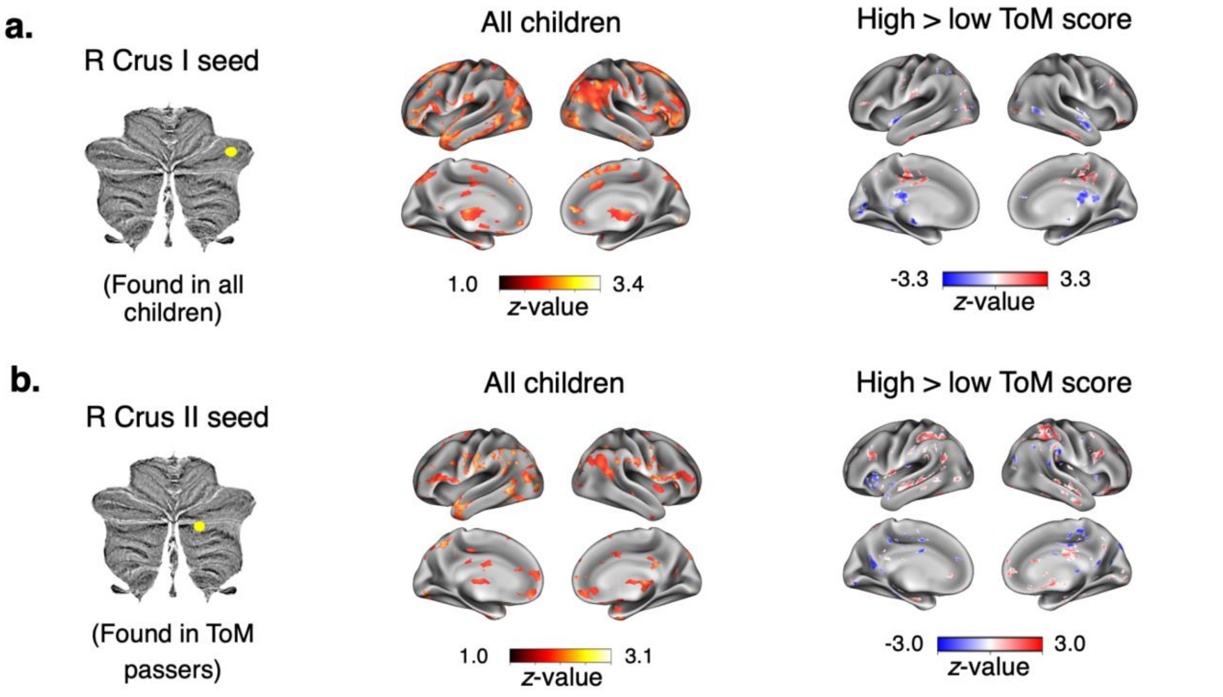

Supplementary figure 8. Seed-to-voxel functional connectivity between cerebellar ToM clusters and the cerebral cortex in an independent dataset (BCP; Howell et al, 2019; N = 26) (z-scored and FDR-corrected at q = .05). **a.** Left: One-sample t-test showing connectivity of right (r) Crus I (cluster found in all children in the Richardson et al. data) with the cerebral cortex in the whole sample of children. Right: GLM of connectivity as a function of children's ToM (CSUS) score for rCrus I. **b.** Left: One-sample t-test showing connectivity of rCrus II (cluster found in ToM passers in the Richardson et al. data) with the cerebral cortex in all children. Right: GLM of connectivity as a function of children's ToM (CSUS) score for rCrus II. All analyses are two-sided.

Supplementary table 1

MNI coordinates of ToM ROIs in all children

ROI	MNI Coordinates		
	x	y	z
Cerebral cortex			
vmPFC	4	51	-10
PreC	0	-46	36
rTPJ	43	-65	38
lTPJ	-48	-65	38
Cerebellum			
rCrus I	52	-68	-26
rIX	2	-46	-44
lIX	-8	-46	-44

Abbreviations: vmPFC = ventromedial prefrontal cortex; PreC = precuneus; r/lTPJ = right/left temporoparietal junction.

Supplementary table 2

MNI coordinates of ToM ROIs in ToM passers

ROI	MNI Coordinates		
	**x**	**y**	**z**
Cerebral cortex			
dmPFC	-6	45	42
vmPFC	4	56	0
PreC	14	-48	30
rTPJ	42	-64	40
lTPJ	-48	-67	37
Cerebellum			
rCrus I	53	-56	-30
lCrus I	-30	-84	-30
rCrus II	22	-87	-36
rIX	8	-50	-44
lIX	-8	-52	-38

Abbreviations: dmPFC = dorsomedial prefrontal cortex; vmPFC = ventromedial prefrontal cortex; PreC = precuneus; r/lTPJ = right/left temporoparietal junction.

Supplementary table 3

MNI coordinates of ToM ROIs in ToM non-passers

ROI	MNI Coordinates		
	x	y	z
Cerebral cortex			
vmPFC	2	55	-11
PreC	14	-55	26
rTPJ	42	-67	38
lTPJ	-34	-72	43
Cerebellum			
rCrus I	54	-68	-30
rIX	3	-44	-44
lIX	-8	-44	-44

Abbreviations: vmPFC = ventromedial prefrontal cortex; PreC = precuneus; r/lTPJ = right/left temporoparietal junction.

Supplementary table 4

Number of participants with ToM ROIs defined at p > .05

**Group**		**rTPJ**		**lTPJ**		**dmPFC**		**vmPFC**		**PreC**		**rCrus II**		**lCrus II**		**rCrus I**		**lCrus I**	
		***p*<.1**	***p*<1**	***p*<.1**	***p*<1**	***p*<.1**	***p* <1**	***p*<.1**	***p*<1**	***p*<.1**	***p*<1**	***p*<.1**	***p*<1**	***p*<.1**	***p*<1**	***p*<.1**	***p*<1**	***p*<.1**	***p*<1**
Adults	***N***																		
Richardson et al. (2018)	22	0	0	0	0	1	0	1	1	0	1	1	0	1	0	-	-	-	-
Caltech Conte Center	56	0	0	0	0	2	0	1	1	0	0	2	3	0	3	-	-	-	-
Children	***N***																		
All	41	0	0	1	0	-	-	3	1	3	0	-	-	-	-	3	9	-	-
ToM passers	22	0	0	0	0	1	0	0	0	0	0	7	1	-	-	4	4	3	4
ToM non-passers	19	0	0	1	0	-	-	3	1	2	0	-	-	-	-	3	9	-	-

ROIs were identified based on the ToM > pain movie contrast (two-sided *t*-test, uncorrected; see **Methods: Dynamic causal modeling**). Dashes (-) represent ROIs that were not applicable in a given sample. *N* refers to the total number of participants in a given sample. Abbreviations: dmPFC = dorsomedial prefrontal cortex; vmPFC = ventromedial prefrontal cortex; PreC = precuneus; r/lTPJ = right/left temporoparietal junction.

Supplementary Methods

ToM specificity

We performed additional exploratory analyses to ensure that the observed differences in functional activations between ToM passers and non-passers (**Figure 1**) were specific to children's ToM abilities and not driven by the development of general cognitive abilities or other cognitive functions. First, we ran a second-level general linear model (GLM) on the entire developmental sample, including children's ToM score (ratio predictor: 0-6) as well as children's IQ. Given that children performed a different out-of-scanner IQ test depending on their age (see **Behavioral task battery**), we added two IQ predictors in our model: *IQ test type*, a binary categorical variable indicating which IQ test a child completed (WPPSI or KBIT-II), and *IQ score*, a continuous variable including the pooled standardized scores of the two IQ tests. The effect of ToM score on functional activation in the cerebellum (**Figure 1b**) was similar to the main results in bilateral medial Crus I (rCrus I: 19 -85 -30; lCrus I: -40 -75 -35) and Crus II (rCrus II: 42 -74 -45; lCrus II: -12 -69 -43) ($p_{uncorr.} < .001$, FDR-corrected: $q = .05$; **Supplementary figure 1**). It should be noted that IQ demonstrated a relatively high positive correlation with ToM score (Spearman's $r(39) = .72$, $p < .001$).

Additionally, we examined if functional activation in the cerebellum for bodily pain was also affected by children's out-of-scanner ToM task performance. This would mean that the functional differences between ToM passers and non-passers were not necessarily driven by ToM abilities, but some other process (e.g., simply reflected changes due to age). On a single-subject level, we followed the method described in **Contrast analyses**, but focused on the pain > ToM contrast (as opposed to the ToM > pain contrast). On the group level, we performed two separate one-sample *t*-tests in the groups of ToM passers and non-passers ($p_{uncorr.} < .001$, FDR-corrected: $q = .05$). We observed functional clusters in the bilateral IV that did not overlap with the clusters found for the ToM > pain contrast **(Figure 1c-d**), both in ToM passers (rIV: 25 -71 -23; lIV: -34 -73 -24) and non-passers (rIV: 26 -74 -22; lIV: -29 -74 -22), further ensuring the ToM specificity of our results (**Supplementary figure 3**).

Age and sex correction

We performed additional exploratory analyses with age and sex as covariates, to ensure that the observed results are not driven by further individual differences. Age in particular was relatively highly correlated with ToM score (two-sided Spearman's $r(39) = .72$, $p = 9.94 \times 10^{-8}$, Cohen's $d = 2.07$, 95% CI = [0.53, 0.84]; see **Figure 1g**), which is expected as both the brain and behavior fundamentally change in development. Nevertheless, we sought to investigate associations that remained significant over and above the effect of age in models where we directly contrasted children's ToM abilities. We first performed a GLM to assess ToM activation during the movie-watching task as a function of children's ToM abilities, while controlling for age and sex. The effect of ToM score on functional activation in the cerebellum (**Figure 1b**) were largely similar in bilateral medial Crus I (rCrus I: 19 -85 -30; lCrus I: -40 -75 -35) and Crus II (rCrus II: 42 -74 -45; lCrus II: -12 -69 -43) ($p_{uncorr.} < .001$, FDR-corrected: $q = .05$; **Supplementary figure 2**).

We then compared cerebro-cerebellar connectivity between ToM passers and non-passers by repeating our seed-to-voxel functional connectivity two-sample *t*-tests for rCrus I and rCrus II while controlling for age and sex. As in our main analyses, ToM non-passers demonstrated greater connectivity with regions not overlapping with the cerebral ToM network for the rCrus I seed (identified in all children) ($p_{uncorr.} < .001$, FDR-corrected: $q = .05$; **Supplementary figure 4a**). Conversely, ToM passers demonstrated greater connectivity with clusters of the ToM network, namely the TPJ and STS, for the rCrus II seed (identified in ToM passers), consistent with our main findings ($p_{uncorr.} < .001$, FDR-corrected: $q = .05$; **Supplementary figure 4b**). However, these connectivity differences were smaller than the ones observed in our main findings. Additionally, connectivity between the rCrus II and the PreC, a core node of the cerebral ToM network, seemed to unexpectedly decrease in ToM passers, contrary to our main findings (**Supplementary figure 4b)**. This discrepancy could be at least partially attributed to the high collinearity between age and ToM score, which could obscure the true relationship between connectivity and ToM abilities, making it difficult to disentangle the effects of these two variables. Future studies should further investigate this by examining the mechanisms of cerebro-cerebellar connectivity in ToM development.

Functional connectivity replication analysis

Data source. We validated our functional connectivity analyses in resting-state data from an independent dataset to ensure that the results were indeed driven by cerebro-cerebellar connectivity and not correlations that arise from the movie stimulus itself. We leveraged openly available data from the Baby Connectome Project (BCP; Howell et al., 2019). The BCP contains structural, resting-state functional, and behavioral data of healthy term born infants from birth to 5 years of age. For consistency with the Richardson et al. sample, and to ensure that the ToM emergence period at ~3 years is fully covered (Grosse Wiesmann et al., 2020), we focused on children between 2-5 years of age, whose ToM abilities were scored based on the CSUS (Tahiroglu et al., 2014). We visually inspected the quality of T1w and resting-state data for motion or other artifacts. As in Richardson et al. (2018), we additionally defined artifact timepoints as timepoints displaying >2 mm composite motion relative to a previous timepoint or timepoints where global signal was over 3 SD relative to all participants' mean global signal after preprocessing. Based on these quality checks, we excluded participants with poor scan quality and excessive motion artifacts (over one third of timepoints). This led to a final sample of 26 children (age: *M* (*SD*) = 2.86 (0.95) years; 15 female).

Data acquisition. Data acquisition parameters are described in more detail elsewhere (Howell et al., 2019). Briefly, data were acquired with 3T Siemens Prisma MRI scanners (Siemens, Erlangen, Germany) with 32 channel head coils. Younger participants were scanned while naturally asleep without the use of sedatives. T1w images were acquired with a 3D MPRAGE sequence and the following parameters: sagittal FOV of 256 × 240 mm with a matrix size of 320 × 300 × 208 slices, resolution of 0.8 mm isotropic voxels, flip angle of 8 degrees, and TR/TE parameters of 2400/2.24 ms. Resting-state BOLD images were acquired in two phase-encoding directions (AP-PA) and the following parameters: sagittal FOV of 208 × 208 mm with a matrix size of 104 × 91 × 72 slices, resolution of 2 mm isotropic voxels, flip angle of 52 degrees, and TR/TE parameters of 800/37 ms.

CSUS score. The CSUS (Tahiroglu et al., 2014) is a 42-item parental questionnaire that assesses mental state understanding in young children. The measure has proven reliable and valid in assessing early emerging ToM abilities, including beliefs about other people's mental states, such as desires (e.g., differences between what people desire and what they get) and beliefs (e.g., understanding that lying can mislead other people). CSUS scores strongly correlate with out-of-

scanner ToM tasks (Tahiroglu et al., 2014). Scores range between 1 and 4, with higher scores demonstrating better ToM abilities.

Preprocessing and data analysis. We preprocessed T1w and resting-state BOLD data with NiBabies, a robust preprocessing pipeline optimized for infants and young children (Goncalves et al., 2022). T1w images underwent RAS reorientation, intensity correction, skull-stripping, tissue segmentation, and normalization to a pediatric MNI template. BOLD images were corrected for motion and aligned to the normalized T1w images. Additional nuisance confounds were calculated, including composite motion parameters and fluctuations in global signal. For consistency with the Richardson et al. analyses, we included the number of artifact timepoints (see **Data source**) and PCA-derived CompCor noise regressors as confounds in all first-level analyses. Our seed-to-voxel connectivity analyses followed the same procedure as in the main analyses in the Richardson et al. dataset. Specifically, we used the cerebellar ToM clusters from the Richardson et al. sample as 5 mm spheres to calculate connectivity between the cerebellum and all voxels in the cerebral cortex on a single-subject level. For each cerebellar ROI, we then performed two-sided group-level one-sample *t*-tests to test significant connections against zero and assessed connectivity as a function of CSUS score with a GLM, in which we included children's CSUS score as a continuous predictor (in all analyses: $p < .001$ uncorrected; FDR threshold: $q = .05$).

Results. Results were largely consistent with the ones observed in the main analyses. Specifically, as in the main analyses, the rCrus I seed, identified in all children from the Richardson et al. (2018) sample, was correlated with regions of the cerebral ToM network (e.g., TPJ, STS, dmPFC, vmPFC), as well as regions that do not typically belong to that network (e.g., dorsolateral PFC, cingulate, and thalamus) (group-level one-sample *t*-test, $p_{uncorr.} < .001$, FDR-corrected: $q = .05$; **Supplementary figure 8a, left**). GLM analyses with CSUS score as a continuous predictor demonstrated that connectivity with the non-ToM cerebral regions decreased, whereas connectivity with the PreC of the cerebral ToM network increased as a function of increasing CSUS scores ($p_{uncorr.} < .001$, FDR-corrected: $q = .05$; **Supplementary figure 8a, right**). As in the main analyses, the rCrus II seed, which was only found in ToM passers in the Richardson et al. sample, demonstrated more specific connections with the TPJ, STS, PreC, dmPFC, and vmPFC of the cerebral ToM network, and fewer connections with non-ToM regions than the rCrus I (group-level one-sample *t*-test, $p_{uncorr.} < .001$, FDR-corrected: $q = .05$; **Supplementary figure 8b, left**). These connections with the cerebral ToM network were more prominent as CSUS scores

increased, as evidenced by a GLM analysis with CSUS score as a continuous predictor ($p_{uncorr.} < .001$, FDR-corrected: $q = .05$; **Supplementary figure 8b, right**).

Together, results are in line with the increase in specificity with ToM-network connectivity as a function of children's ToM abilities that we found in the main study. However, these results should be interpreted with caution due to the small sample size in these analyses. In particular, even though we found convergent patterns with our main analysis, we also note some discrepancies in the observed clusters, for example less pronounced connectivity with the PreC and more connections with non-ToM regions for both seeds. Future studies should replicate these findings using larger sample sizes.
