# Supplementary material for: Functional recruitment and connectivity of the cerebellum is associated with the emergence of Theory of Mind in early childhood
Source: Nat Commun. 2025 Jun 6;16:5273. doi: 10.1038/s41467-025-60523-9 (PMC12144182; doi:10.1038/s41467-025-60523-9)
Supplement: Supplementary file 2 — Reporting Summary [file 41467_2025_60523_MOESM2_ESM.pdf]

nature portfolio

Corresponding author(s): Aikaterina Manoli, Sofie L. Valk

Last updated by author(s): Apr 15, 2025

Reporting Summary

Nature Portfolio wishes to improve the reproducibility of the work that we publish. This form provides structure for consistency and transparency in reporting. For further information on Nature Portfolio policies, see our Editorial Policies and the Editorial Policy Checklist.

Statistics

For all statistical analyses, confirm that the following items are present in the figure legend, table legend, main text, or Methods section.

n/a	Confirmed	
☐	☒	The exact sample size (*n*) for each experimental group/condition, given as a discrete number and unit of measurement
☐	☒	A statement on whether measurements were taken from distinct samples or whether the same sample was measured repeatedly
☐	☒	The statistical test(s) used AND whether they are one- or two-sided<br>*Only common tests should be described solely by name; describe more complex techniques in the Methods section.*
☐	☒	A description of all covariates tested
☐	☒	A description of any assumptions or corrections, such as tests of normality and adjustment for multiple comparisons
☐	☒	A full description of the statistical parameters including central tendency (e.g. means) or other basic estimates (e.g. regression coefficient) AND variation (e.g. standard deviation) or associated estimates of uncertainty (e.g. confidence intervals)
☐	☒	For null hypothesis testing, the test statistic (e.g. *F*, *t*, *r*) with confidence intervals, effect sizes, degrees of freedom and *P* value noted<br>*Give P values as exact values whenever suitable.*
☐	☒	For Bayesian analysis, information on the choice of priors and Markov chain Monte Carlo settings
☒	☐	For hierarchical and complex designs, identification of the appropriate level for tests and full reporting of outcomes
☐	☒	Estimates of effect sizes (e.g. Cohen's *d*, Pearson's *r*), indicating how they were calculated

Our web collection on statistics for biologists contains articles on many of the points above.

Software and code

Policy information about availability of computer code

Data collection	N/A
Data analysis	Nilearn (0.10.0) in Python 3.9.5. SPM12 (including SUIT 3.5) in MATLAB R2022b. All code is available at github.com/kmanoli/DevCerebToM.

For manuscripts utilizing custom algorithms or software that are central to the research but not yet described in published literature, software must be made available to editors and reviewers. We strongly encourage code deposition in a community repository (e.g. GitHub). See the Nature Portfolio guidelines for submitting code & software for further information.

Data

Policy information about availability of data

All manuscripts must include a data availability statement. This statement should provide the following information, where applicable:

- Accession codes, unique identifiers, or web links for publicly available datasets
- A description of any restrictions on data availability
- For clinical datasets or third party data, please ensure that the statement adheres to our policy

All raw and preprocessed data for this study are available on OpenNeuro (Richardson et al.: https://openneuro.org/datasets/ds000228/versions/1.1.0; CCC: https://openneuro.org/datasets/ds003798/versions/1.0.5).

Research involving human participants, their data, or biological material

Policy information about studies with human participants or human data. See also policy information about sex, gender (identity/presentation), and sexual orientation and race, ethnicity and racism.

Reporting on sex and gender	Biological sex was collected via self-report. Informed consent for reporting was obtained by participants or participants' guardians if they were underage. We included 41 children (25 biological female) children and 78 adults (36 biological female). We included biological sex as a covariate in supplementary exploratory analyses. Findings apply to both sexes.
Reporting on race, ethnicity, or other socially relevant groupings	We did not include or report such variables. Such data was only collected in one of the datasets we used (Kliemann et al., 2022) via self-report. We did not consider such data in our analyses.
Population characteristics	We included a sample of typically developing children (age range: 3-12 years; M (SD) age = 5.91 (2.29) years) and neurotypical adults (M (SD) age = 27.68 (6.79) years).
Recruitment	Details about recruitment can be found in the original study manuscripts: Richardson et al. (2018) and Kliemann et al. (2022).
Ethics oversight	Datasets come from open sources and have been approved by the associated ethics committees. Our institution did not require additional additional permission to use this data.

Note that full information on the approval of the study protocol must also be provided in the manuscript.

Field-specific reporting

Please select the one below that is the best fit for your research. If you are not sure, read the appropriate sections before making your selection.

☐ Life sciences ☒ Behavioural & social sciences ☐ Ecological, evolutionary & environmental sciences

For a reference copy of the document with all sections, see nature.com/documents/nr-reporting-summary-flat.pdf

Behavioural & social sciences study design

All studies must disclose on these points even when the disclosure is negative.

Study description	Quantitative (fMRI movie-watching task and out-of-scanner behavioral)
Research sample	We used open data of children (N=41; age range: 3-12 years; M (SD) age = 5.91 (2.29) years; 25 female) and adults (N=78; M (SD) age = 27.68 (6.79) years; 36 female). We chose this data because it wad perfectly tailored to our research purpose, which was to examine the role of the cerebellum in early-life ToM emergence and compare it to its role in adult ToM.
Sampling strategy	All participants were recruited via convenience sampling. Adults in Kliemann et al., 2022 were recruited from Los Angeles via Craigslist or flyers. Children and adults in Richardson et al., 2018 were recruited via advertisements in the local community. Please refer to the associated manuscripts for more details.
Data collection	Adults were scanned in a 3T MRI scanner. Children were scanned in a 3T MRI scanner holding a stuffed animal for comfort and an experimenter was present to ensure they were awake and attentive during scanning. Children additionally performed an out-of-scanner false-belief assessment in which an experimenter told a story and children had to answer questions about characters in the story. Responses were collected with pen and paper. Please refer to the associated manuscripts for more details.
Timing	There was no designated start and stop of data collection. Please refer to the associated manuscripts for more details.
Data exclusions	Participants in Kliemann et al., 2022 were excluded if they had an IQ below 90, no English proficiency, any mood or neurodevelopmental disorders, vision or hearing impairments, or were born prematurely. This led to the exclusion of 66 participants in the original study. We further excluded 2 participants for poor fMRI data quality, as indexed by three independent raters' visual examination of preprocessed data in the original study. Thus, our sample consisted of 56 adults.<br><br>Participants in Richardson et al., 2018 were excluded if they did not complete all behavioral and imaging components, moved excessively in the scanner, or had language delays. his led to the exclusion of 19 participants in the original study. We further excluded 71 children and 8 adults with incomplete cerebellar coverage. Thus, our sample consisted of 41 children 1and 22 adults.<br><br>Please refer to the associated manuscripts for more details.
Non-participation	12 children dropped out from the original Richardson et al. 2018 study and 8 adults from the original Kliemann et al. 2022 study. Please refer to the associated manuscripts for more details.
Randomization	There were no experimental groups and thus no randomization. All participants watched the same in-scanner movie and all children completed the same out-of-scanner ToM assessment.

Reporting for specific materials, systems and methods

We require information from authors about some types of materials, experimental systems and methods used in many studies. Here, indicate whether each material, system or method listed is relevant to your study. If you are not sure if a list item applies to your research, read the appropriate section before selecting a response.

Materials & experimental systems

n/a	Involved in the study
☒	☐ Antibodies
☒	☐ Eukaryotic cell lines
☒	☐ Palaeontology and archaeology
☒	☐ Animals and other organisms
☒	☐ Clinical data
☒	☐ Dual use research of concern
☒	☐ Plants

Methods

n/a	Involved in the study
☒	☐ ChIP-seq
☒	☐ Flow cytometry
☐	☒ MRI-based neuroimaging

Plants

Seed stocks: *Report on the source of all seed stocks or other plant material used. If applicable, state the seed stock centre and catalogue number. If plant specimens were collected from the field, describe the collection location, date and sampling procedures.*

Novel plant genotypes: *Describe the methods by which all novel plant genotypes were produced. This includes those generated by transgenic approaches, gene editing, chemical/radiation-based mutagenesis and hybridization. For transgenic lines, describe the transformation method, the number of independent lines analyzed and the generation upon which experiments were performed. For gene-edited lines, describe the editor used, the endogenous sequence targeted for editing, the targeting guide RNA sequence (if applicable) and how the editor was applied.*

Authentication: *Describe any authentication procedures for each seed stock used or novel genotype generated. Describe any experiments used to assess the effect of a mutation and, where applicable, how potential secondary effects (e.g. second site T-DNA insertions, mosiacism, off-target gene editing) were examined.*

Magnetic resonance imaging

Experimental design

Design type: Movie-watching task

Design specifications: All participants watched a 5.6min movie which included ToM and bodily pain scenes. Seven ToM and nine pain event timepoints (ToM: 60 s total, M (SD) length: 8.6 (4.6) s, pain: 66 s total, M (SD) length: 7.3 (4.4) s were defined in the original Richardson et al. (2018) study.

Behavioral performance measures: Children in Richardson et al. 2018 performed an out-of-scanner false-belief assessment, where they answered six questions about the mental states of the characters in a story.

Acquisition

Imaging type(s): Functional and structural (T1w)

Field strength: 3T

Sequence & imaging parameters: Richardson et al. 2018: T1-weighted images were acquired in 176 interleaved sagittal slices with 1 mm isotropic voxels [GRAPPA parallel imaging, acceleration factor of 3; FOV: 256 mm (adult coil); 192 mm (children coils)]. Functional data were collected with a gradient-echo EPI sequence in 32 interleaved near-axial whole-brain slices aligned with the anterior/posterior commissure (EPI factor: 64; TR: 2 s, TE: 30 ms, flip angle: 90°).

Kliemann et al., 2022: T1-weighted structural data were acquired with 0.9 mm isotropic voxels (multi-echo MEMP-RAGE pulse sequence; acceleration factor of 2; flip angle: 7°; water excite fat suppression). Functional data were collected with a multi-band 2.5 mm isotropic T2*-weighted EPI sequence (EPI echo spacing: 0.49 ms; TR: 0.7 s, TE: 30 ms, flip angle: 53°).

Area of acquisition: Whole brain (incl. cerebellum)

Diffusion MRI: ☐ Used ☒ Not used

Preprocessing

Preprocessing software

We used preprocessed structural and functional data which had undergone robust motion artifact removal and quality control in the original datasets. Briefly, functional data in Richardson et al.'s study were registered to the first image of the run, co-registered to each participant's anatomical image, and the anatomical image was normalized to the ICBM/MNI 152 2009c Nonlinear Asymmetric space (MNI152NLin2009cAsym) template. Data were then smoothed with a 5 mm Gaussian kernel. Functional and structural data in the original CCC dataset were processed with a multi-step pipeline, including fMRIPrep 20.2.161. In summary, fMRIPrep processing involved registration of functional images to a reference volume and co-registration to each participant's anatomical image, slice-timing correction and resampling into the MNI152NLin2009cAsym standard space. Here, we additionally applied a 5 mm Gaussian kernel to the preprocessed data for consistency with the Richardson et al. data.

Normalization

Nonlinear registration of functional and structural images to template

Normalization template

Cerebral cortex: ICBM152. Cerebellum: SUIT

Noise and artifact removal

In Richardson et al., 2018, denoising involved motion artifact detection via the ART toolbox (https://www.nitrc.org/projects/artifact_detect/), where artifacts were defined as timepoints displaying >2 mm composite motion relative to a previous timepoint or timepoints where global signal was over 3 SDs relative to all participants' mean global signal. Additionally, five principal component analysis (PCA)-based noise regressors were generated via CompCor within subject-specific white matter masks. In Kliemann et al., 2022, we also focused on PCA-derived CompCor regressors defined in the original dataset for denoising the functional data: six CompCor regressors were calculated within the intersection of the subcortical mask, the cerebrospinal fluid and the white matter mask for each participant.

Volume censoring

N/A

Statistical modeling & inference

Model type and settings

Contrast analyses: First, whole-brain ToM activations were identified via the ToM scenes > bodily pain scenes contrast on an individual-subject level ($p < .001$, uncorrected). The level-one statistical maps were then passed on to separate group-level one-sample t-tests (two-sided; $p < .001$, uncorrected) for children and each of the adult samples to test for the significance of ToM activations in the ToM > pain contrast against zero. In children, we also performed a level-two general linear model (GLM) with children's ToM score (0-6) as a ratio predictor to investigate differences in cerebellar activations as a function of ToM abilities (two-sided; $p < .001$, uncorrected). All analyses were FDR-corrected at $q = .05$.

Seed-to-voxel connectivity: We correlated the BOLD signal within each of the cerebellar ToM ROIs and every voxel in the cerebral cortex for every participant. Single-subject seed-to-voxel correlation matrices (corrected for nuisance regressors, as above) were passed on to a group-level two-sided one-sample t-test to identify significant seed-to-voxel functional connections against zero ($p < .001$ uncorrected; FDR threshold: $q = .05$). We conducted separate one-sample t-tests for each adult sample, the whole developmental sample, ToM passers, and ToM non-passers. Lastly, we directly compared functional connectivity between ToM passers and ToM non-passers, between adults and ToM passers, as well as adults and ToM non-passers with separate level-two independent t-tests (two-sided; $p < .001$ uncorrected; FDR threshold: $q = .05$).

Dynamic causal modeling: In all analyses, we specified a full bilinear deterministic DCM on a single-subject level, without centering around the mean, which included: i) all forward and backward fixed (or endogenous) connections between the ROIs; ii) all modulatory connections which reflected connectivity changes due to the ToM condition; iii) direct input parameters which reflected input driving connectivity between ROIs in both the ToM and pain conditions, by combining onsets of both conditions in a single vector. On the group level, we constructed a parametric empirical Bayes (PEB) model over all connectivity parameters, which makes it possible to estimate the group-average effective connectivity while taking within-participant variability into account. A PEB was also chosen because it allowed us to control for individual differences between participants (e.g., passing or failing the ToM test) by including them as a covariate in some of our models. Last, we used Bayesian model reduction to automatically remove connectivity parameters that did not contribute to the model evidence from the group-level PEB model, by performing a greedy search. Connectivity parameters whose posterior probability was over $p > .95$ were considered significant. Note that following a Bayesian approach in the single-subject DCM and group-level PEB estimations circumvents the multiple comparisons problem.

Effect(s) tested

1) Percent BOLD signal change in the cerebellum in ToM scenes during an in-scanner movie. 2) Changes in functional and effective connectivity between ToM ROIs in the cerebellum and the cerebral cortex.

Specify type of analysis: ☐ Whole brain ☐ ROI-based ☒ Both

Anatomical location(s)

In adults, ToM ROIs were defined based on seminal studies that guided previous functional and effective connectivity ToM studies on cerebro-cerebellar ToM (King et al., 2019; Van Overwalle et al., 2019). In children, we used a different approach since there are no studies that have identified ToM regions in the developing cerebellum. ROIs were centered around group local maxime for the ToM > pain contrast in the movie-watching task ($p < .001$, uncorrected; FDR: $q = .05$; $k = 5$). We used the same approach for the cerebral cortex for consistency.

Statistic type for inference

(See Eklund et al. 2016)

Contrast analyses were performed voxel-wise, functional connectivity was computed by correlating seed time series with the time series of all other voxels, and effective connectivity was computed parcel-wise (between every pair of ROIs).

Correction | FDR (q = .05)

Models & analysis

n/a	Involved in the study
☐	☒ Functional and/or effective connectivity
☒	☐ Graph analysis
☒	☐ Multivariate modeling or predictive analysis

Functional and/or effective connectivity | See "Model type and settings" above.
